# Association between Vitamin D Receptor Gene Polymorphisms with Childhood Temporal Lobe Epilepsy

**DOI:** 10.3390/ijerph121113913

**Published:** 2015-10-30

**Authors:** Pei Jiang, Wen-Ye Zhu, Xin He, Mi-Mi Tang, Rui-Li Dang, Huan-De Li, Ying Xue, Li-Hong Zhang, Yan-Qin Wu, Ling-Juan Cao

**Affiliations:** 1 Institute of Clinical Pharmacy and Pharmacology, Second Xiangya Hospital, Central South University, Changsha 410011, China; E-Mails: jpoet89@126.com (P.J.); zhuwenyewawbmg@126.com (W.-Y.Z.); hexin526@126.com (X.H.); tangmimi1989@126.com (M.-M.T.); ruilidang@gmail.com (R.-L.D.); xueying091@126.com (Y.X.); zhanglihong0612@163.com (L.-H.Z.); wuyanqin1992@163.com (Y.-Q.W.); caolingjuan@126.com (L.-J.C.); 2 Department of Pharmacy, Jining First People’s Hospital, Jining 272011, China

**Keywords:** epilepsy, children, vitamin D receptor, polymorphisms

## Abstract

Vitamin D (VD) is implicated in multiple aspects of human physiology and vitamin D receptor (*VDR*) polymorphisms are associated with a variety of neuropsychiatric disorders. Although VD deficiency is highly prevalent in epilepsy patients and converging evidence indicates a role for VD in the development of epilepsy, no data is available on the possible relationship between epilepsy and genetic variations of *VDR*. In this study, 150 controls and 82 patients with temporal lobe epilepsy (TLE) were genotyped for five common *VDR* polymorphisms (Cdx-2, FokI, BsmI, ApaI and TaqI) by the polymerase chain reaction-ligase detection reaction method. Our results revealed that the frequency of FokI AC genotype was significantly higher in the control group than in the patients (*p* = 0.003, OR = 0.39, 95% CI = 0.21–0.73), whereas the AA genotype of ApaI SNP was more frequent in patients than in controls (*p* = 0.018, OR = 2.92, 95% CI = 1.2–7.1). However, no statistically significant association was found between Cdx-2, BsmI and TaqI polymorphisms and epilepsy. Additionally, in haplotype analysis, we found the haplotype GAT (BsmI/ApaI/TaqI) conferred significantly increased risk for developing TLE (*p* = 0.039, OR = 1.62, 95% CI = 1.02–2.56). As far as we know, these results firstly underline the importance of *VDR* polymorphisms for the genetic susceptibility to epilepsy.

## 1. Introduction

Epilepsy is one of the most prevalent neurological disorders of childhood, affecting six out of every 1000 children, and it is about twice as common in children as in adults [1]. Temporal lobe epilepsy (TLE) is the most frequent form among epilepsies with focal seizure on set and displays resistance to antiepileptic drugs [2]. In approximately 20%–30% of cases, there is a clear extraneous acquired cause (e.g., head trauma, stroke), but in the remainder, genetic factors play a major role. Indeed, numerous association studies have shown the involvement of various genes in TLE and it is now believed that TLE is influenced by genetic variations as well as environmental factors [3,4].

Over the last decade, vitamin D (VD) is increasingly recognized as a neuroactive steroid modulating brain development and function in addition to its classical role in calcium-phosphate homeostasis and bone health. Multiple lines of evidence indicate that VD is implicated in numerous brain processes including regulating neurotrophic factors, neuroimmunomodulation and neurotransmission [5,6,7]. Poor VD status and genetic variations of vitamin D receptor (*VDR*) are implicated in the pathogenesis of a number of neuropsychiatric disorders including Alzheimer’s disease, depression and multiple sclerosis [8,9,10]. However, regarding epilepsy, while VD deficiency is frequently observed in children with epilepsy, most of the studies in this field focused on the association of VD deficiency with the reduced bone mineral density, but not with epilepsy itself. Recently, a pilot study found that correcting the deficient VD status can improve seizure control in epilepsy patients [11]. Additionally, animal studies also demonstrated that VD treatment can raise the electroconvulsive threshold in rats [12], and on the other hand, *VDR* mutant mice displays increased susceptibility to chemically induced seizures [13]. Considering that certain genetic variations of *VDR* may have regulatory effect on VD signaling and metabolism, it is tempting to hypothesize that the particular polymorphisms of *VDR* could contribute to the development of epilepsy or subgroups of this condition, notably TLE. Therefore, we performed a genetic association analysis of five known *VDR* SNPs Cdx-2 (rs11568820), FokI (rs2228570), BsmI (rs1544410), ApaI (rs7975232) and TaqI (rs731236) in TLE patients and controls.

## 2. Materials and Methods

### 2.1. Subjects

A total of 82 unrelated Asian patients (39 females and 43 males; age range, 2–15 years; Han Chinese from Hunan Province) were included in this study. Patients with epilepsy were recruited at the outpatient clinic of the Second Xiangya Hospital. The patients were diagnosed and classified according to the guidelines of the International League Against Epilepsy. In each patient, the diagnosis of TLE was made on the basis of a range of clinical seizure semiology, EEG and MRI criteria that are considered to be reliable interictal indicators of TLE. The diagnosis of TLE was mainly based on typical temporal auras or ictal and interictal EEG discharges over the temporal lobes in the presence of focal spikes or sharp waves followed by slow waves. Clinical MRI examinations followed a protocol routinely used for patients with TLE, which briefly included high-resolution T1-weighted volume acquisition and T2-weighted and fluid-attenuated inversion recovery (FLAIR) sequences [14]. Brain MRI evaluation revealed evidence of hippocampal sclerosis in eight of the 82 patients. MRI did not detect any mass lesions such as tumors, cortical dysgenesis, vascular lesions, malformations or post-traumatic scars in any of the patients. None of them had mental retardation, psychiatric difficulties or early psychiatric manifestations. The mean age at seizure onset was 6.8 ± 3.17 years (range 0.25–12.72 years) and the mean duration of epilepsy was 2.23 ± 1.91 years (range 0–7.70 years). Six of the 82 patients had family histories of seizures or febrile convulsions in one or more first- to third-degree relatives. We also enrolled 150 healthy control subjects, 19 to 60 years of age, matched for sex and ethnicity, without a history of seizures, including febrile convulsions, related family histories or inherited central nerve system diseases. Written informed consent was signed by each participant or responsible adult before they participated in the study. The study was conducted in accordance with the Declaration of Helsinki, and the protocol was approved by the Ethics Committee of the Second Xiangya Hospital of Central South University (S209. 2012).

### 2.2. DNA Isolation and Genotyping

Genomic DNA was extracted from 1 mL of venous blood by the using SQ Blood DNA Kit II (D0714–250, OMEGA, Norcross, GA, USA) according to the manufacturer’s instructions. The genotype of SNPs was analyzed using the polymerase chain reaction‑ligase detection reaction (PCR-LDR) method. The primers for both PCR and LDR reactions were all designed by the Primer3 online software v.0.4.0 (http://frodo.wi.mit.edu/) and are shown in Table 1. These PCR products and the LDR probes were then subjected to a multiplex LDR reaction, with a DNA sequencer used to detect the products. To test the validity of this procedure, approximately 10% of the samples were randomly selected and retested using the same procedure. The products generated in the retested samples were consistent with those obtained in the original sample.

**Table 1:** Primers of target genes used in the PCR.

SNP	Ancestor Allele	Primer Sequence	Product Size
Cdx-2 (rs11568820)	A	5’-CATCTTTTGTATCAGGAAC-3’ (forward) 5’-AACTGCAACCCATAATAAG-3’ (reverse)	103 bp
FokI (rs2228570)	A	5’-TGGCCTGCTTGCTGTTCTTA-3’ (forward) 5’-AAGTCTCCAGGGTCAGGCA-3’ (reverse)	92 bp
BsmI (rs1544410)	G	5’-AGCCCAGTTCACGCAAGAG-3’ (forward) 5’-TAGATAAGCAGGGTTCCTGG-3’ (reverse)	100 bp
ApaI (rs7975232)	C	5’-TTGAGTGTCTGTGTGGGTGG-3’ (forward) 5’-TTAGAGAAGAAGGCACAGGAG-3’ (reverse)	99 bp
TaqI (rs731236)	T	5’-TTCTCTATCCCCGTGCCCA-3’ (forward) 5’-TTGGACAGGCGGTCCTGGAT-3’ (reverse)	84 bp

### 2.3. Statistical Analysis

The Hardy-Weinberg equilibrium was assessed for every SNP in the controls by Chi-square test (χ2 test). Haplotype analysis was performed using the online SHEsis software at http://analysis.bio-x.cn (Shanghai, China). Odds ratio (OR) and the 95% confidence interval (CI) were calculated using the unconditional logistic regression analysis to evaluate the association between each SNP and the risk of TLE with adjustment for age and gender. All the results were analyzed using the SPSS version 13.0 software (SPSS Inc., Chicago, IL, USA). The two-sided *p* values of less than 0.05 were considered statistically significant.

## 3. Results

### 3.1. Genotype and Allele Frequencies of the VDR Polymorphisms

The genotype and allele frequencies of all five *VDR* SNPs in the two groups are shown in Table 2. Each SNP was examined with Hardy-Weinberg equilibrium among controls and no significant difference was observed. The frequency of FokI AC genotype was found to be significantly higher in the control group than in the patients (*p* = 0.003, OR = 0.39, 95% CI = 0.21–0.73), indicating a potential association with neuroprotection. 

**Table 2:** Genotype and allele distribution of the *VDR* polymorphisms among cases and controls and the association with the risk of epilepsy.

SNP	Genotype/Allele	Cases *n* (%)	Controls *n* (%)	OR (95% CI)	*p* Value
Cdx-2	GG	28 (34.1)	44 (29.3)		(1.095–3.281)
(rs11568820)	AG	39 (47.6)	73 (48.7)	0.84 (0.46–1.56)	0.58
	AA	15 (18.3)	33 (22.0)	0.71 (0.33–1.55)	0.39
	G	95 (57.9)	161(53.7)		
	A	69 (47.1)	139 (46.3)	1.19 (0.81–1.75)	0.38
FokI	CC	32 (39.0)	33 (22.0)		
(rs2228570)	AC	34 (41.5)	86 (57.3)	0.39 (0.21–0.73)	0.003
	AA	16 (19.5)	31 (20.1)	0.52 (0.24–1.12)	0.095
	A	66 (40.2)	148 (49.3)		
	C	98 (59.8)	152 (50.7)	1.446 (0.98–2.22)	0.06
BsmI	GG	72 (87.8)	133 (88.7)		
(rs1544410)	AG	7 (8.5)	16 (10.7)	0.81 (0.32–2.10)	0.66
	AA	3 (3.7)	1 (0.7)	5.54 (0.57–54.25)	0.14
	G	151 (92.1)	282 (94.0)		
	A	13 (7.9)	18 (6.0)	1.35(0.64–2.83)	0.43
ApaI	CC	41 (50.0)	79 (52.7)		
(rs7975232)	AC	26 (31.7)	61 (40.7)	0.82 (0.15–1.49)	0.52
	AA	15 (18.3)	10 (6.7)	2.92 (1.20–7.14)	0.018
	C	108 (65.9)	219 (73.0)		
	A	56 (34.1)	81 (27.0)	1.40 (0.93–2.12)	0.11
TaqI	TT	72 (87.8)	127 (84.7)		
(rs731236)	CT	6 (7.3)	21 (14.0)	0.51 (0.19–0.31)	0.16
	CC	4 (4.9)	2 (1.3)	3.53 (0.63–19.74)	0.15
	T	150 (91.5)	275 (91.7)		
	C	14 (8.5)	25 (8.3)	1.03 (0.52–2.03)	0.94

Abbreviations: CI, confidence interval; OR, odds ratio.

Meanwhile, the AA genotype of ApaI SNP was more frequent in cases than in controls suggesting an association with increased susceptibility to TLE (*p* = 0.018, OR = 2.92, 95% CI = 1.2–7.1). However, there was no statistically significant difference between patients and controls for the genotype and allele distributions of Cdx-2, BsmI and TaqI polymorphisms.

### 3.2. Haplotype Analysis

In accordance with previous findings [15], BsmI, ApaI and TaqI were found to be in strong linkage disequilibrium (BsmI/ApaI: D’ = 0.797, r2 = 0.279; BsmI/TaqI: D’ = 0.676, r2 = 0.356; ApaI/TaqI: D’ = 0.835, r2 = 0.223). Haplotype frequencies are shown in Table 3. The haplotype analysis revealed that haplotype GAT significantly increased the risk of TLE in childhood (*p* = 0.039, OR = 1.62, 95% CI = 1.02–2.56). This effect was not detected in any other common haplotypes.

**Table 3:** Haplotype frequencies for *VDR* polymorphisms in cases and controls.

Haplotype (BsmI/ApaI/TaqI)	Cases 2*n* = 164 (%)	Controls 2*n* = 300 (%)	OR (95% CI)	*p* Value
AAC	7.98 (4.9)	12.78 (4.3)	1.19 (0.48–2.94)	0.71
GAC	2.02 (1.2)	12.22 (4.1)	0.30 (0.07–1.36)	0.10
GAT	41.98 (25.6)	54.65 (18.2)	1.62 (1.02–2.56)	0.039
GCT	104.00 (63.4)	215.13 (71.7)	0.74 (0.49–1.13)	0.16

Abbreviations: CI, confidence interval; OR, odds ratio. Haplotypes were omitted if the estimated haplotype frequency was <3%.

## 4. Discussion

This work firstly showed the potential association of *VDR* polymorphisms and *VDR* haplotypes with the susceptibility to TLE among Chinese children. Genetic variations of *VDR* are known to have a significant effect on VD signaling and the five mostly studied SNPs (Cdx-2, FokI, BsmI, ApaI and TaqI) have been linked to an increased risk of a variety of diseases. FokI polymorphism is located inside the translation initiation codon of *VDR* and if this SNP contains C allele, an alternative start side is used, resulting in a three amino acid shorted protein, which may lead to altered *VDR* function [16]. Cdx-2 is an A-G transition in the intestine-specific binding site of transcription factor Cdx-2, located in the 5’ promoter region of *VDR*. An allele of this SNP is associated with lower *VDR* promoter activity [17]. The three SNPs located near the 3’ un-translated region (3’UTR) (BsmI, ApaI and TaqI), albeit not functional, are linked with a poly (A) microsatellite repeat in the 3′UTR that could influence the *VDR* mRNA stability [18]. While previous data have linked these SNPs with multiple neurological disorders, the evidence concerning the relationship between *VDR* polymorphisms and epilepsy remains blank.

Deficient VD status is frequently observed in patients with epilepsy [19]. However, due to the increased rates of bone fractures and abnormalities in epilepsy patients, the influence of antiepileptic drugs (AEDs) on VD levels and bone metabolism is the mostly studied aspect of epilepsy and VD [20]. AEDs can induce the VD catabolizing cytochrome P450 enzymes and accelerate the conversion of VD into its inactive metabolites [21] and the *VDR* polymorphisms are associated with the drug-induced reduction in bone mineral density [22]. Interestingly, recent studies indicate that even before AED treatment, VD deficiency is highly prevalent in children with epilepsy [23] and the circulating VD concentrations continue to decrease following months of antiepileptic medication [24]. Considering the varied neurological activity of VD, there exists a possibility that VD deficiency or suboptimal VD signaling may increase the susceptibility to epilepsy. The evidence from clinical VD supplementation studies and animal researches also support the antiepileptic action of VD. Furthermore, the association between *VDR* polymorphisms with TLE found in this study further adds weight to the theory that VD signaling might be involved in the development of epilepsy.

Several lines of evidence indicate that the genetic variations of FokI can affect circulating VD status and are associated with many neuropsychiatric diseases. The FokI A allele was found to be associated with neuroprotection and C allele may increase the risk of several neurological disorders, including Parkinson’s disease, Alzheimer’s disease and multiple sclerosis [25,26,27,28]. Consistent with previous findings, we also found modestly but non-significantly higher frequency of C allele (*p* = 0.06) in patients with epilepsy. Likewise, several studies also showed the association between the SNPs in the 3′end of *VDR* gene (BsmI, ApaI and TaqI) and brain dysfunctions [29,30]. However, in addition to that the AA genotype of ApaI slightly increased the risk of TLE (*p* = 0.018), we did not observe statistically significant differences in the genotype or allele frequencies of BsmI and TaqI between patients and controls, which can be explained by either a lack of effect of these SNPs on TLE or be due to a limited number of cases. Notably, single marker association analysis is sometimes not sufficient in complex diseases, whereas the haplotype-based linkage disequilibrium mapping has become a powerful tool for genetic association studies. In the present study, strong linkage disequilibrium was observed among the three SNPs in the 3’end of *VDR* gene, which confirms previous findings [15], and our data suggest that the haplotype GAT is likely to be a disease-risk haplotype, which may affect RNA splicing, processing and editing. In accordance with our findings, the frequency of this haplotype was also found higher in patients with asthma, cardiovascular disease and obesity [15,30,31,32,33]. Thus, it is becoming increasingly necessary for future studies to examine the effect of these SNPs or haplotypes on the interaction and activity characteristics of *VDR* protein.

Multiple actions of VD are likely to underlie its anticonvulsant effect. Altered γ-aminobutyric acid (GABA) status has been found in the brain tissues of rodents fed with a VD deficient diet [34] and we recently found that chronic administration of 1,25-dihydroxyvitamin D [1,25(OH)_2_D], the active form of VD, can elevate GABA concentrations and promote glutamate decarboxylase (GAD) 67 and GAD65 mRNA expression in rat brain [6], whereas attenuated GABAergic neurotransmission is highly relevant to the progression of epilepsy [35]. Additionally, VD is also implicated as a neuro-immunomodulator and can suppress the proconvulsant inflammatory cytokines [36]. Furthermore, there is evidence that VD can protect the neural cells from excessive unbuffered calcium and reactive oxygen species, the key factors of glutamate excitotoxicity [37].

However, it should be noted that the interpretation of our data is complicated by the puzzling finding that while heterozygous FokI AC genotype seems to increase the resistance to TLE, the two homozygous genotypes had no effect on the susceptibility. These results can be explained by that the relatively small sample size in each genotype has insufficient statistically power to detect a slight effect, which is a major limitation of the present study. Our study is also limited by the failure of controlling for the potential confounding factors, such as sunshine exposure and the intake of VD and calcium. Additionally, we also failed to analyze the serum VD status and functional consequence of these genetic variations. Considering that the interactions between various genes and (or) environmental factors play a role in the actions of *VDR*, the association between *VDR* polymorphisms and TLE is likely to be confounded by the various potential gene-gene and (or) gene-environmental interactions. Thus, future studies are needed to further determine the effects of these SNPs on circulating VD status or the expression of the key components of VD signaling in the brain tissues or the peripheral blood mononuclear cells of seizure patients, which would lend more support to the relationship between VD and epilepsy.

## 5. Conclusions

In conclusion, we found a significant association between *VDR* genetic variations and the risk of TLE in a Chinese Han population. Our data provide new evidence for the involvement VD in the development of epilepsy, which should promote replication studies in ethnically disparate populations in cohort study samples and with variants covering the whole gene.
